# Shunt and right ventricular structural findings in isolated anomalous pulmonary venous return in Turner syndrome

**DOI:** 10.1186/1532-429X-15-S1-P296

**Published:** 2013-01-30

**Authors:** Laura Olivieri, Anjali Chelliah, Li-Yueh Hsu, Richard B Thompson, Vladimir Bakalov, Douglas Rosing, Carolyn Bondy, Andrew E Arai

**Affiliations:** 1 National Heart, Lung and Blood Instutie, The National Institutes of Health, Bethesda, MD, USA; 2 Cardiology, Children's National Medical Center, Washington, DC, USA; 3 Biomedical Engineering, University of Alberta, Edmonton, AB, Canada; 4 National Institute of Child Health and Development, Section on Epigenetics and Development, The National Institutes of Health, Bethesda, MD, USA

## Background

Isolated anomalous pulmonary venous return of one pulmonary vein is relatively rare (~1/1000) and has an unclear natural history. Patients with Turner syndrome (TS) have a relatively high incidence of partial anomalous pulmonary venous return (PAPVR). Our aim is to describe the natural history of unrepaired isolated anomalous pulmonary venous return and its hemodynamic consequences through analysis of cardiac volumetric data and Qp:Qs ratios in asymptomatic volunteers with TS.

## Methods

249 subjects underwent cardiac magnetic resonance imaging in an IRB-approved natural history study of TS at the National Institutes of Health. Cardiac anatomy was characterized with steady-state free precession cine imaging, phase contrast imaging, as well as contrast enhanced magnetic resonance angiograms in adults. Fifteen patients with partial anomalous pulmonary venous return and 42 age-matched controls with TS and normal pulmonary venous return were identified. Ventricular functional analysis was performed by a pediatric cardiologist. Phase offsets on velocity encoded phase contrast images were corrected using 3-dimensional fits to static pixels.

## Results

Subjects ranged in age from 9-62, with a mean age of 36.3 years. One of fifteen patients with PAPVR was found to have two anomalous pulmonary veins, an RVEDVi of 83 mL/m2 and a Qp:Qs ratio of 1.8. She underwent surgical correction. The remaining fourteen patients all had isolated PAPVR. The median right ventricular end diastolic volume (RVEDV) indexed to body surface area (BSA) of the PAPVR group was 78 (IQR 57-94) ml/m2 and the normal TS group was 64 (49-72) ml/m2 (p = 0.023). Amongst the group of patients with PAPVR, the median Qp:Qs ratio was 1.56 (1.3-1.8). Based on cine MRI volumetric analysis, the median ratio of RV:LV stroke volume was 23% higher in the TS with PAPVR group compared with the normal TS group (p = 0.001)

## Conclusions

Women and girls with TS and isolated anomalous pulmonary venous return have larger right ventricles than age matched controls that are, however, within the normal range for indexed RV volumes. The median Qp:Qs estimates by velocity-encoded phase contrast are slightly greater than the usual threshold for consideration of surgical intervention. These observations demonstrate cardiovascular changes in patients with isolated PAPVR that should be monitored.

## Funding

National Institutes of Health National Heart, Lung and Blood Institute Intramural Funding

